# Proteolytic shedding of the prion protein via activation of metallopeptidase ADAM10 reduces cellular binding and toxicity of amyloid-β oligomers

**DOI:** 10.1074/jbc.RA118.005364

**Published:** 2019-03-14

**Authors:** Heledd H. Jarosz-Griffiths, Nicola J. Corbett, Helen A. Rowland, Kate Fisher, Alys C. Jones, Jennifer Baron, Gareth J. Howell, Sally A. Cowley, Satyan Chintawar, M. Zameel Cader, Katherine A. B. Kellett, Nigel M. Hooper

**Affiliations:** From the ‡ Division of Neuroscience and Experimental Psychology, School of Biological Sciences, Faculty of Biology, Medicine and Health, AV Hill Building, University of Manchester, Manchester Academic Health Science Centre, Oxford Road, Manchester M13 9PT,; the § Flow Cytometry Facility Laboratory, Faculty of Biology, Medicine, and Health, University of Manchester, CTF Building, Oxford Road, Manchester M13 9PT,; the ¶ Sir William Dunn School of Pathology, University of Oxford, South Parks Road, Oxford OX1 3RE,; the ‖ Oxford Parkinson's Disease Centre, University of Oxford, South Parks Road, Oxford OX1 3QX,; the ** Nuffield Department of Clinical Neurosciences, University of Oxford, John Radcliffe Hospital, Oxford OX3 9DU, and; the ‡‡ Weatherall Institute of Molecular Medicine, University of Oxford, John Radcliffe Hospital, Oxford OX1 3QX, United Kingdom

**Keywords:** amyloid-beta (AB), induced pluripotent stem cell (iPS cell) (iPSC), prion, neurodegenerative disease, Alzheimer disease, oxidative stress, cell surface protein, metallopeptidase ADAM10, proteolytic shedding

## Abstract

The cellular prion protein (PrP^C^) is a key neuronal receptor for β-amyloid oligomers (AβO), mediating their neurotoxicity, which contributes to the neurodegeneration in Alzheimer's disease (AD). Similarly to the amyloid precursor protein (APP), PrP^C^ is proteolytically cleaved from the cell surface by a disintegrin and metalloprotease, ADAM10. We hypothesized that ADAM10-modulated PrP^C^ shedding would alter the cellular binding and cytotoxicity of AβO. Here, we found that in human neuroblastoma cells, activation of ADAM10 with the muscarinic agonist carbachol promotes PrP^C^ shedding and reduces the binding of AβO to the cell surface, which could be blocked with an ADAM10 inhibitor. Conversely, siRNA-mediated ADAM10 knockdown reduced PrP^C^ shedding and increased AβO binding, which was blocked by the PrP^C^-specific antibody 6D11. The retinoic acid receptor analog acitretin, which up-regulates ADAM10, also promoted PrP^C^ shedding and decreased AβO binding in the neuroblastoma cells and in human induced pluripotent stem cell (iPSC)-derived cortical neurons. Pretreatment with acitretin abolished activation of Fyn kinase and prevented an increase in reactive oxygen species caused by AβO binding to PrP^C^. Besides blocking AβO binding and toxicity, acitretin also increased the nonamyloidogenic processing of APP. However, in the iPSC-derived neurons, Aβ and other amyloidogenic processing products did not exhibit a reciprocal decrease upon acitretin treatment. These results indicate that by promoting the shedding of PrP^C^ in human neurons, ADAM10 activation prevents the binding and cytotoxicity of AβO, revealing a potential therapeutic benefit of ADAM10 activation in AD.

## Introduction

Alzheimer's disease (AD)^3^ is a progressive, age-associated disorder that is characterized by abnormal accumulation of proteinacious aggregates in the form of β-amyloid (Aβ) containing plaques and neurofibrillary tangles composed of hyperphosphorylated tau (1, 2). Oligomers of Aβ (AβOs) appear to be the most neurotoxic species, binding to receptors on the surface of neurons and triggering a variety of downstream signaling pathways that negatively impact neuronal function and survival (reviewed in Refs. 3 and 4). A substantial portion of AβO toxicity in AD is mediated following the initial interaction with the cellular prion protein (PrP^C^), which resides in cholesterol-rich lipid rafts at the neuronal surface (4, 5). AβO binding to PrP^C^ mediates inhibition of long-term potentiation in hippocampal slices (6) and memory and behavioral impairments in multiple AD mouse models (7, 8). The binding of AβO to PrP^C^ leads to activation of Fyn kinase, a loss of surface *N*-methyl-d-aspartate receptors, and subsequent phosphorylation of tau (9–11). AβO also cause increases in reactive oxygen species (ROS), which contribute to the neurodegeneration (reviewed in Ref. 12).

Given the central role of PrP^C^ in mediating the toxicity of AβO, targeting PrP^C^ has potential for AD therapy (reviewed in Ref. 4). Immunotargeting (*e.g.* using the anti-PrP^C^ mAb 6D11 to block the AβO binding site on PrP^C^) prevented the impairment in long-term potentiation caused by AβO derived from AD brain extracts (13, 14) and blocked Aβ synaptotoxicity following peripheral administration (15). Altering the conformation of AβO, disrupting AβO binding to PrP^C^, or displacing PrP^C^ from lipid rafts blocked downstream cellular toxicity (11, 16). Several of the actions of AβO, including activation of Fyn, dendritic spine loss, and tau phosphorylation, are mediated by PrP^C^ coupling to mGluR5 (17–19), and pharmacological inhibition or allosteric modulation of mGluR5 reduced pathogenesis in AD mouse models (20, 21). Another approach has been to target Fyn directly with a specific inhibitor to rescue the memory deficits in an AD mouse model (22). These approaches highlight that targeting PrP^C^ or other components of the AβO-PrP^C^ signaling complex may have therapeutic potential in AD.

Aβ peptides are generated when the amyloid precursor protein (APP) is cleaved by the sequential action of the β-secretase (β-site APP-cleaving enzyme 1; BACE1) and the multisubunit γ-secretase complex in the amyloidogenic pathway (23). β-Secretase cleavage of APP also releases the large soluble ectodomain fragment sAPPβ. Alternatively, APP can be cleaved via the nonamyloidogenic pathway through the action of the α-secretase, a disintegrin and metalloprotease ADAM10, precluding the formation of the Aβ peptide and generating an alternative soluble fragment sAPPα that has neuroprotective and neurotrophic properties (23). It is generally assumed that there is competition between the α- and β-secretases for their substrate APP, resulting in a reciprocal relationship between the amyloidogenic and nonamyloidogenic APP-processing pathways. In support of this reciprocal relationship, neuronal overexpression of ADAM10 in APP_V717I_ transgenic mice increased the secretion of sAPPα and reduced the formation of Aβ peptides (24), whereas in human induced pluripotent stem cell (iPSC)-derived neurons, inhibition of BACE1 reduced sAPPβ and Aβ and increased sAPPα (25).

The ectodomain shedding of multiple cell surface proteins can be promoted by a variety of compounds. For example, activators of protein kinase C and the muscarinic agonist carbachol promote the shedding of APP (26–29). The vitamin A analog, acitretin, promoted the α-secretase cleavage of APP by stimulating the transcription of ADAM10 via interaction with retinoic acid–responsive elements within the *ADAM10* promoter (30). As ADAM10 also cleaves and sheds the ectodomain of PrP^C^ from the cell surface (31–33), we hypothesized that modulating ADAM10 activity, thereby altering the shedding and thus the amount of PrP^C^ at the cell surface, would modulate the binding and toxicity of AβO.

Here, we have used human neuroblastoma cells and iPSC-derived cortical neurons to show that carbachol and acitretin promote the shedding of cell surface PrP^C^ through activation of ADAM10. The resulting reduction of cell surface PrP^C^ leads to a concomitant reduction in the binding of AβO. Conversely, siRNA knockdown of ADAM10 resulted in increased cell surface PrP^C^ and a corresponding increase in AβO binding that could be blocked with the PrP^C^ antibody, 6D11. AβO binding to PrP^C^ activated Fyn kinase and caused an increase in ROS that could be blocked by promoting the shedding of PrP^C^ with acitretin. We also report that although acitretin reciprocally modulated the amyloidogenic and nonamyloidogenic processing of APP in neuroblastoma cells and rat hippocampal neurons, no such reciprocal relationship was observed in the human iPSC-derived neurons.

## Results

### Promoting the shedding of PrP^C^ decreases the cell surface binding of AβO

**Figure 1.**
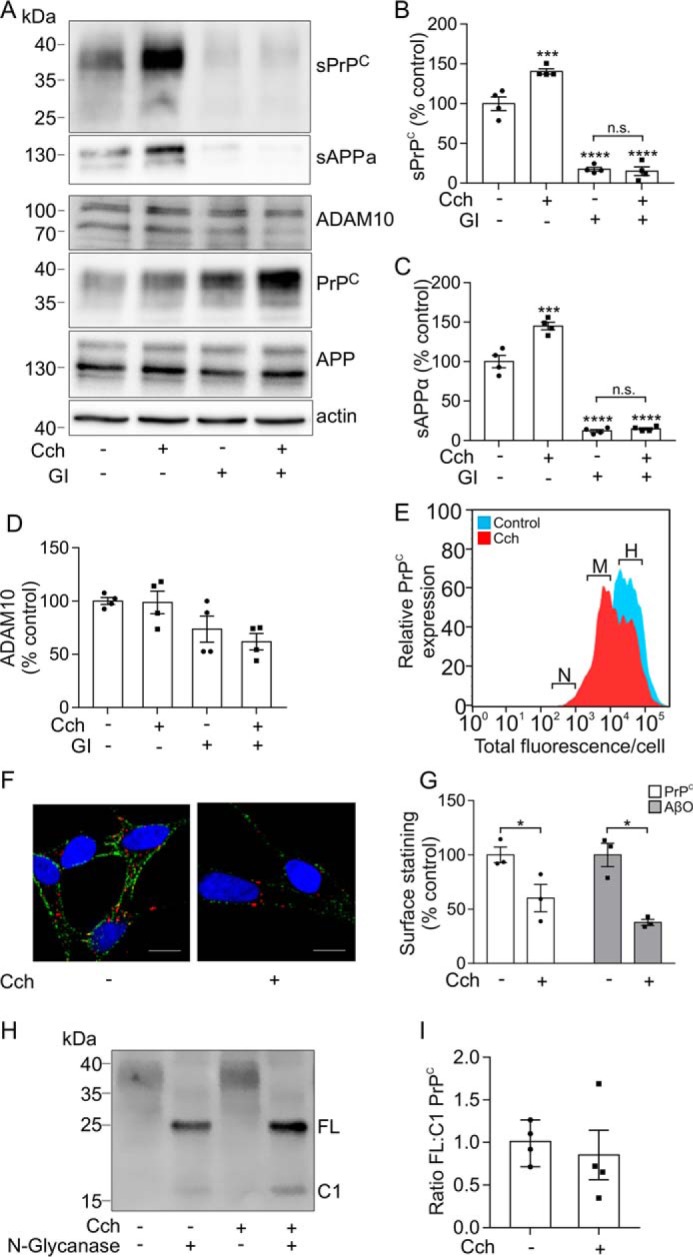
**Promoting the shedding of PrP^C^ decreases AβO binding in an ADAM10-dependent manner.** *A*, immunoblots of sPrP^C^ and sAPPα in concentrated conditioned media, and of ADAM10, PrP^C^, APP, and actin in cell lysates, from SH-SY5Y cells expressing PrP^C^ incubated with or without carbachol (*Cch*; 20 μm) and with or without GI254023X (*GI*; 10 μm) in Opti-MEM for 24 h at 37 °C. sPrP^C^ (*B*), sAPPα (*C*), and ADAM10 (*D*) immunoblots were quantified and are represented as a percentage of control (*n* = 4). *E*, ImageStream imaging cytometry analysis showing negative (*N*), middle (*M*), and high (*H*) levels of cell surface PrP^C^ expression in SH-SY5Y cells expressing PrP^C^ incubated without (*blue*) and with (*red*) carbachol (20 μm). *F*, immunofluorescence microscopy images showing staining of PrP^C^ (*green*) and Aβ-biotin (*red*) in nonpermeabilized SH-SY5Y cells expressing PrP^C^ incubated with or without carbachol (20 μm) in Opti-MEM for 3 h at 37 °C followed by incubation with AβO (500 nm) for 30 min at room temperature. *Scale bar*, 5 μm. *G*, quantification of PrP^C^ cell surface staining (*n* = 3) and AβO cell surface binding (*n* = 3). *H*, immunoblot of PrP^C^ in cell lysate samples, prepared from SH-SY5Y cells expressing PrP^C^ incubated with or without carbachol (20 μm), and treated without or with *N*-glycanase for 16 h at 37 °C. *I*, quantification of deglycosylated full-length (*FL*) and C1 PrP^C^ species, expressed as the ratio of FL/C1 (*n* = 4). Statistical analyses were one-way ANOVA with Tukey's post hoc correction for multiple-comparison data or independent *t* test with Welch's correction for two-sample comparison. Data are shown as mean ± S.E. (*error bars*); *n.s.*, not significant; *, *p* < 0.05; ***, *p* < 0.001; ****, *p* < 0.0001.

As ADAM10 mediates the shedding of PrP^C^ from the cell surface (31, 32), we hypothesized that activation of ADAM10 would reduce AβO binding to cells due to shedding of its cell surface receptor PrP^C^. Initially, the muscarinic agonist carbachol, which increases the shedding of multiple cell surface proteins, including APP, was employed (28). The effect of carbachol on PrP^C^ and APP shedding was monitored by detection of the soluble fragments, sPrP^C^ and sAPPα, respectively, in the media fraction. Treatment of human SH-SY5Y cells expressing PrP^C^ with carbachol promoted the shedding of full-length glycosylated PrP^C^ by 1.4-fold and the α-secretase cleavage of APP by 1.5-fold (Fig. 1, *A–C*). To establish that carbachol was acting via activation of ADAM10, the cells were incubated with the ADAM10-selective inhibitor, GI254023X (34). On its own, the ADAM10 inhibitor significantly reduced the amount of sPrP^C^ in the media by 83% (Fig. 1, *A* and *B*), consistent with ADAM10 being the major constitutive PrP^C^ sheddase (32). In the presence of the ADAM10 inhibitor, carbachol failed to cause an increase in the shedding of PrP^C^ (Fig. 1, *A* and *B*), indicating that carbachol was promoting the shedding of PrP^C^ via activation of ADAM10. Similar to its effect on the shedding of PrP^C^, the ADAM10 inhibitor significantly reduced sAPPα in the media by 85% and blocked the increase in sAPPα caused by carbachol (Fig. 1, *A* and *C*). Treatment with carbachol and the GI254023X (*GI*) inhibitor did not significantly alter ADAM10 expression (Fig. 1*D*). Consistent with increasing the shedding of PrP^C^, carbachol treatment resulted in a decrease in cell surface expression of PrP^C^ evaluated by ImageStream imaging cytometry (Fig. 1*E*). This analysis demonstrated a decrease in high-expressing PrP^C^ cells in a population of cells treated with carbachol when compared with the control population. The decrease in cell surface PrP^C^ was confirmed using immunofluorescence microscopy, where carbachol treatment of the cells caused a significant decrease (40%) in the amount of PrP^C^ localized to the cell surface (Fig. 1, *F* and *G*). AβOs were prepared and characterized as described previously (11), and their binding to the cells was monitored by immunofluorescence microscopy. Treatment of the cells with carbachol resulted in a significant decrease (62%) in the amount of AβO bound to the cell surface (Fig. 1, *F* and *G*).

In addition to promoting the shedding of the entire ectodomain of PrP^C^ through cleavage near the site of glycosylphosphatidylinositol (GPI) anchor attachment (31), ADAM10 has also been reported to cleave PrP^C^ within the middle of the ectodomain (sometimes referred to as α-cleavage) (35). The α-cleavage of PrP^C^ releases an N-terminal fragment, termed N1, and leaves a C-terminal fragment, C1, tethered to the membrane via the GPI anchor. To establish whether carbachol also promoted the cleavage of PrP^C^ within its ectodomain, samples were deglycosylated so that the C1 fragment could be detected and distinguished from unglycosylated full-length PrP^C^. There was no change in the ratio of full-length PrP^C^ to C1 fragment when cells were incubated with carbachol (Fig. 1, *H* and *I*), indicating that carbachol reduces AβO binding by promoting the shedding of full-length PrP^C^ and not by promoting cleavage within its ectodomain.

### Knockdown of ADAM10 increases Aβ oligomer binding in a PrP-dependent manner

**Figure 2.**
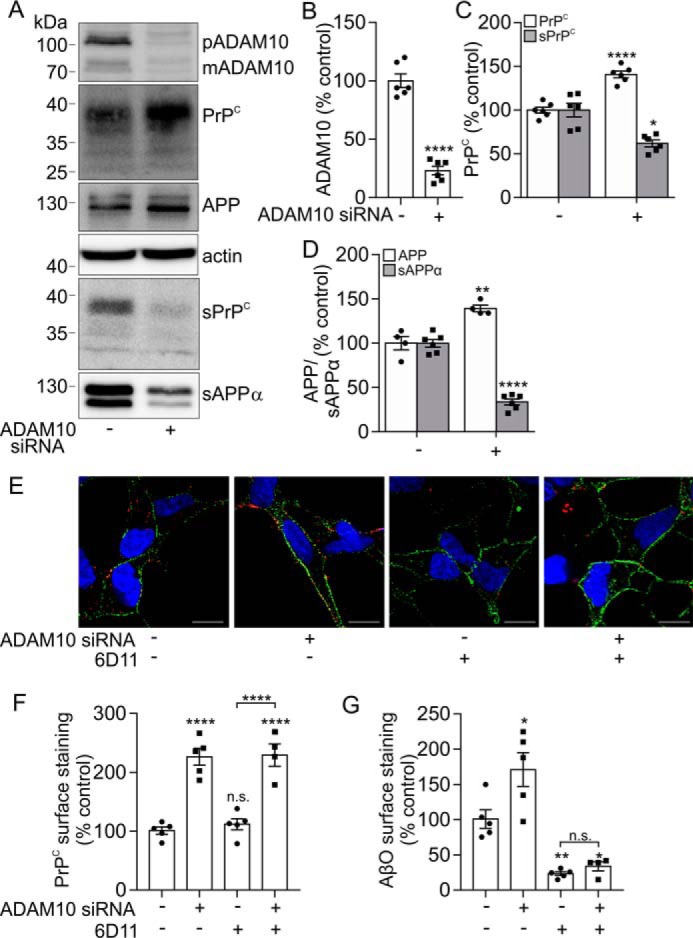
**Knockdown of ADAM10 reduces the shedding of PrP^C^ and increases AβO binding.** *A*, immunoblots of ADAM10 (pro- (*p*) and mature (*m*) forms), PrP^C^, APP, and actin in cell lysates and of sPrP^C^ and sAPPα in concentrated conditioned media from SH-SY5Y cells expressing PrP^C^ incubated with either siRNA targeted against ADAM10 (+) (Dharmacon, SMARTpool) or a nontargeting (−) siRNA control for 48 h followed by incubation with Opti-MEM for a further 24 h. ADAM10 (*B*), PrP^C^ and sPrP^C^ (*B*), and APP and sAPPα (*C*) immunoblots were quantified and are represented as a percentage of control (*n* = 4–6). *E*, immunofluorescence microscopy images showing staining of PrP^C^ (*green*) and Aβ-biotin (*red*) in nonpermeabilized SH-SY5Y cells expressing PrP^C^ following siRNA treatment targeted against ADAM10 and incubation in the absence or presence of the PrP^C^ antibody, 6D11, for 20 min at 37 °C followed by AβO incubation (500 nm) for 30 min at room temperature. *Scale bar*, 5 μm. *F*, quantification of PrP^C^ cell surface staining (*n* = 5); *G*, AβO binding to cells (*n* = 5). Statistical analyses were one-way ANOVA with Tukey's post hoc correction for multiple-comparison data or independent *t* test with Welch's correction for two-sample comparison. Data are shown as mean ± S.E. (*error bars*). *n.s.*, not significant; *, *p* < 0.05; **, *p* < 0.01; ****, *p* < 0.0001.

**Figure 3.**
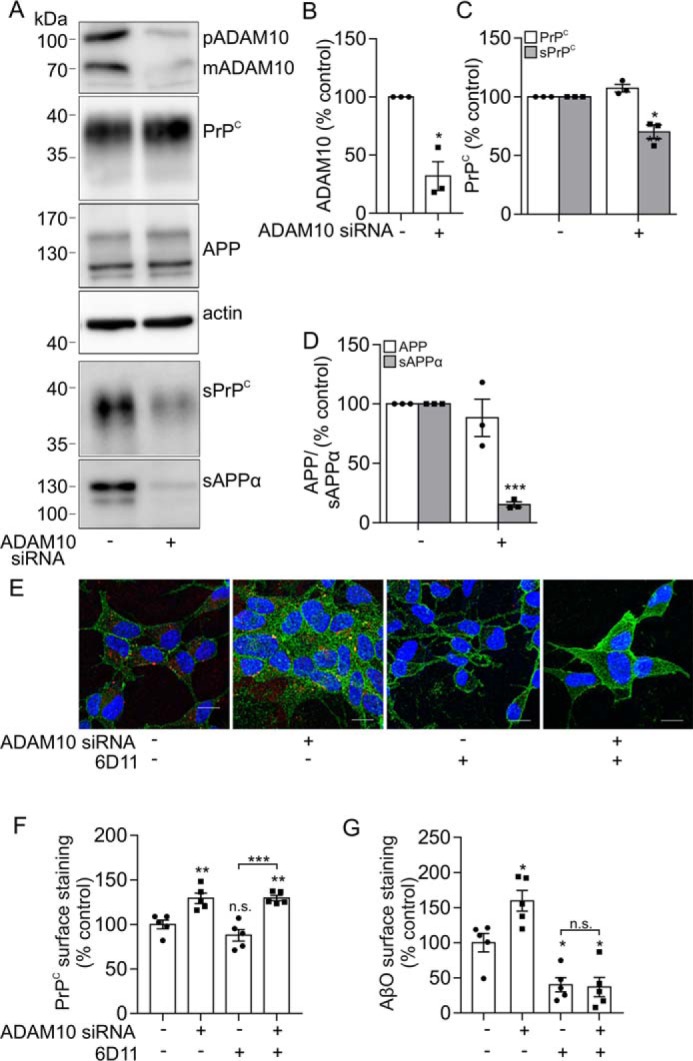
**Knockdown of ADAM10 with an independent siRNA sequence reduces the shedding of PrP^C^ and increases AβO binding.** *A*, immunoblots of ADAM10 (pro- (*p*) and mature (*m*) forms), PrP^C^, APP, and actin in cell lysates and of sPrP^C^ and sAPPα in concentrated conditioned media from SH-SY5Y cells expressing PrP^C^ incubated with either siRNA targeted against ADAM10 (+) (Ambion, single siRNA) or a nontargeting (−) siRNA control for 48 h, followed by incubation with Opti-MEM for a further 24 h. ADAM10 (*B*), PrP^C^ and sPrP^C^ (*C*), and APP and sAPPα (*D*) immunoblots were quantified and are represented as a percentage of control (*n* = 3). *E*, immunofluorescence microscopy images showing staining of PrP^C^ (*green*) and Aβ-biotin (*red*) in nonpermeabilized SH-SY5Y cells expressing PrP^C^ following siRNA treatment targeted against ADAM10 and incubation in the absence or presence of the PrP^C^ antibody, 6D11, for 20 min at 37 °C followed by AβO incubation (500 nm) for 30 min at room temperature. *Scale bar*, 5 μm. Shown are quantification of PrP^C^ cell surface staining (*n* = 5) (*F*) and AβO binding to cells (*n* = 5) (*G*). Statistical analyses were one-way ANOVA with Tukey's post hoc correction for multiple-comparison data or independent *t* test with Welch's correction for two-sample comparison. Data are shown as mean ± S.E. (*error bars*). *n.s.*, not significant; *, *p* < 0.05; **, *p* < 0.01; ***, *p* < 0.001.

To confirm that ADAM10 was responsible for altering AβO binding through modulating the cell surface level of PrP^C^, ADAM10 was knocked down using siRNA in the SH-SY5Y cells expressing PrP^C^. For these experiments, we used both a SMARTpool siRNA containing four target siRNAs (Dharmacon) shown in Fig. 2 and a single, independent siRNA (Ambion) shown in Fig. 3. In the ADAM10 siRNA-treated cells, there was a significant reduction in the pro and mature forms of ADAM10 of 69% (Fig. 2, *A* and *B*) and 68% (Fig. 3, *A* and *B*) in Dharmacon-treated and Ambion siRNA–treated cells, respectively. Following ADAM10 siRNA knockdown, the amount of sPrP^C^ was significantly reduced by 48% (Fig. 2, *A* and *C*) and by 30% (Fig. 3, *A* and *C*), and the amount of sAPPα was also significantly reduced, by 67% (Fig. 2, *A* and *D*) and 85% (Fig. 3, *A* and *D*). ADAM10 siRNA caused a 2.2-fold (Fig. 2, *E* and *F*) and a 1.3-fold (Fig. 3, *E* and *F*) increase in cell surface PrP^C^ relative to nontargeting siRNA as assessed by immunofluorescence microscopy, and the binding of AβO to the cells was increased by 1.7-fold (Fig. 2, *E* and *G*) and 1.6-fold (Fig. 3, *E* and *G*) in the presence of ADAM10 siRNA relative to nontargeting siRNA. To establish whether this increased binding of AβO was due to the increased cell surface level of PrP^C^, cells were preincubated with the PrP^C^ antibody, 6D11, which blocks the AβO binding site on PrP^C^ (6). Incubation of the cells with the 6D11 antibody had no significant effect on the amount of PrP^C^ at the cell surface (Figs. 2 (*E* and *F*) and 3 (*E* and *F*)). However, the 6D11 antibody significantly reduced AβΟ binding to the cells by 77% (Fig. 2, *E* and *G*) and by 60% (Fig. 3, *E* and *G*). In the presence of the 6D11 antibody, siRNA knockdown of ADAM10 failed to increase AβO binding (Figs. 2 (*E* and *G*) and 3 (*E* and *G*)), indicating that the increased binding of AβO upon ADAM10 knockdown was due to increased cell surface PrP^C^.

**Figure 4.**
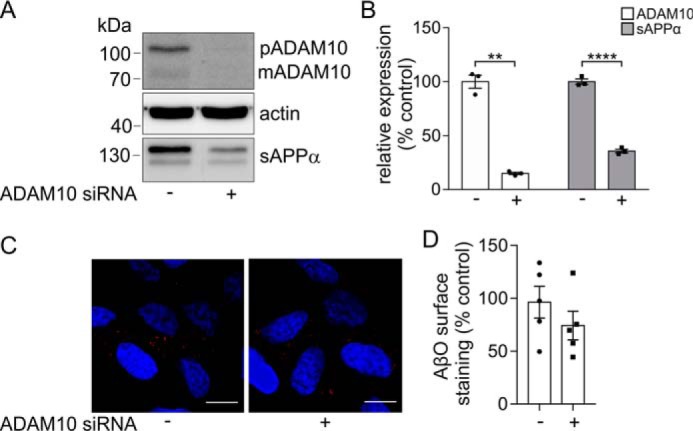
**ADAM10 fails to increase AβO binding in cells lacking PrP^C^.** Immunoblots of ADAM10 and actin in cell lysates and of sAPPα in concentrated conditioned media from untransfected SH-SY5Y cells, which lack endogenous PrP^C^, incubated with either siRNA targeted against ADAM10 (+) or a nontargeting (−) siRNA control for 48 h followed by incubation with Opti-MEM for a further 24 h. *B*, ADAM10 and sAPPα immunoblots were quantified and are represented as a percentage of control (*n* = 3). *C*, immunofluorescence microscopy images showing staining of Aβ-biotin (*red*) in nonpermeabilized untransfected SH-SY5Y cells following siRNA treatment targeted against ADAM10 and incubation with AβO (500 nm) for 30 min at room temperature. *Scale bar*, 5 μm. *D*, quantification of AβO binding to cells (*n* = 5). Statistical analyses were independent *t* test with Welch's correction for two-sample comparison. Data are shown as mean ± S.E. (*error bars*). **, *p* < 0.01; ****, *p* < 0.0001.

To confirm that ADAM10 modulated AβO binding in a PrP^C^-dependent manner, SH-SY5Y cells lacking PrP^C^ (36) were treated with ADAM10 siRNA, and AβO binding was assessed. In these cells, ADAM10 siRNA treatment significantly reduced the pro and mature forms of ADAM10 and the amount of sAPPα in the media (Fig. 4, *A* and *B*). In the absence of PrP^C^, AβO binding was <15% (Fig. 4*C*) of that observed in cells expressing PrP^C^ (Figs. 2*E* and 3*E*), and this residual binding was unchanged following knockdown of ADAM10 (Fig. 4, *C* and *D*). Together, these data indicate that ADAM10 modulates AβO cell surface binding in a PrP^C^-dependent manner.

### Activation of ADAM10 with acitretin increases the shedding of PrP^C^ and decreases AβO binding

**Figure 5.**
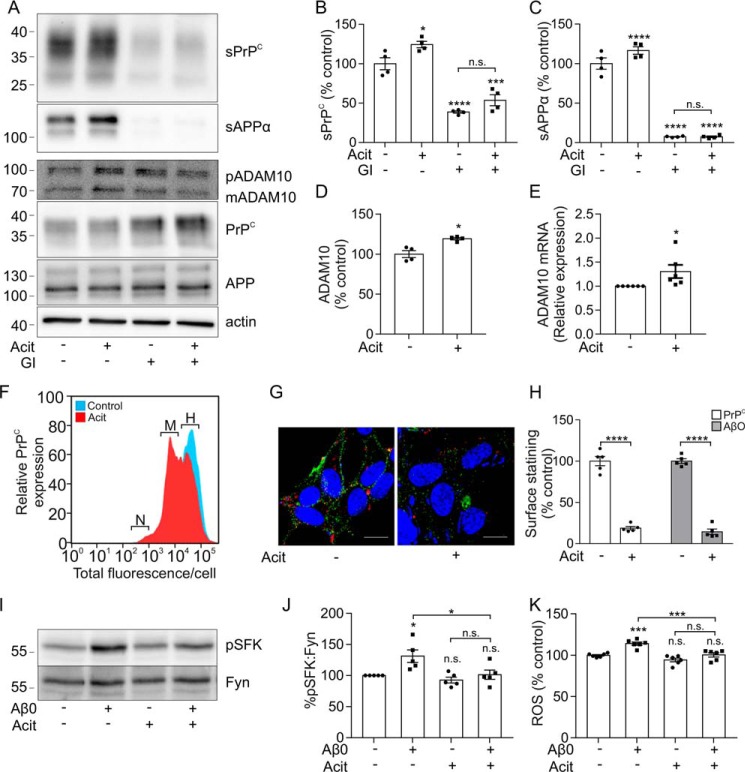
**Acitretin increases PrP^C^ shedding and decreases AβO binding and toxicity in an ADAM10-dependent manner.** *A*, immunoblots of sPrP^C^ and sAPPα in concentrated conditioned media and of ADAM10, PrP^C^, APP, and actin in cell lysates from SH-SY5Y cells expressing PrP^C^ incubated with or without GI254023X (*GI*) (10 μm) and with or without acitretin (*Acit*) (20 μm) diluted in Opti-MEM for 48 h at 37 °C. sPrP^C^ (*B*), sAPPα (*C*), and ADAM10 (*D*) immunoblots were quantified and are represented as a percentage of control (*n* = 4). *E*, relative expression of ADAM10 mRNA in SH-SY5Y cells expressing PrP^C^ incubated with or without acitretin (*Acit*; 20 μm) (*n* = 6). *F*, ImageStream imaging cytometry analysis showing negative (*N*), middle (*M*), and high (*H*) levels of cell surface PrP^C^ expression in SH-SY5Y cells expressing PrP^C^ incubated without (*blue*) and with (*red*) acitretin (20 μm). *G*, immunofluorescence microscopy images showing staining for PrP^C^ (*green*) and Aβ-biotin (*red*) in nonpermeabilized SH-SY5Y cells expressing PrP^C^ incubated with or without acitretin (20 μm) in Opti-MEM for 48 h at 37 °C followed by incubation with AβO (500 nm) for 30 min at room temperature. *Scale bar*, 5 μm. *H*, quantification of PrP^C^ cell surface staining (*n* = 5) and AβO binding to cells (*n* = 5). *I*, immunoblots for the phosphorylated species of Src family kinases (pSFK) and of total Fyn kinase in cell lysates from NB7 cells, which endogenously express PrP^C^, incubated with or without acitretin (20 μm) in Opti-MEM for 48 h at 37 °C followed by incubation with AβO (500 nm) for 20 min at 37 °C. *J*, the ratio of pSFK/Fyn was quantified and is expressed as a percentage of control (*n* = 5). *K*, ROS were measured using the ROS-Glo assay in SH-SY5Y cells expressing PrP^C^ incubated with or without acitretin (20 μm) in Opti-MEM for 48 h at 37 °C and then treated with or without AβO (500 nm) for 90 min at 37 °C. Luminescence was measured and is represented as a percentage of control (*n* = 6). Statistical analyses were one-way ANOVA with Tukey's post hoc correction for multiple-comparison data, or independent *t* test with Welch's correction for two-sample comparison. Data are shown as mean ± S.E. (*error bars*). *n.s.*, not significant; *, *p* < 0.05; ***, *p* < 0.001; ****, *p* < 0.0001.

Acitretin, a synthetic retinoid, increases the expression of ADAM10 in cell culture and animals, leading to an increase in sAPPα (30). As acitretin may have potential as a novel therapeutic drug for AD due to its ability to increase sAPPα in the CSF of AD patients (37), we investigated whether acitretin, via activation of ADAM10, would increase the shedding of PrP^C^ and decrease AβO binding. Acitretin treatment of the SH-SY5Y cells expressing PrP^C^ increased the expression of mature ADAM10 and the shedding of PrP^C^ and sAPPα by 1.2-fold (Fig. 5, *A–C*). In the presence of the ADAM10 inhibitor GI254023X, acitretin failed to induce a significant increase in the amount of sPrP^C^ (Fig. 5, *A* and *B*) or of sAPPα in the media (Fig. 5, *A* and *C*), indicating that it was acting via ADAM10. Treatment with acitretin caused an increase in ADAM10 protein (Fig. 5, *A* and *D*) and mRNA (Fig. 5*E*) expression. Treatment with acitretin resulted in a decrease in cell surface expression of PrP^C^ as evaluated by ImageStream imaging cytometry (Fig. 5*F*). This analysis demonstrated a decrease in high-expressing PrP^C^ cells in a population of cells treated with acitretin when compared with the control population. The decrease in cell surface PrP^C^ was confirmed using immunofluorescence microscopy, where acitretin treatment of the cells caused a significant decrease (81%) in the amount of PrP^C^ localized to the cell surface (Fig. 5, *G* and *H*). This decrease in cell surface PrP^C^ with acitretin resulted in a reduction of the amount of AβO bound to the surface of the cells by 86% (Fig. 5, *G* and *H*). These data indicate that activation of ADAM10 with the retinoic acid analog acitretin increases the shedding of PrP^C^ and decreases the binding of AβO to the cells.

### Activation of ADAM10 prevents AβO-mediated activation of Fyn kinase and rescues AβO-mediated increase in ROS

To establish whether promoting the shedding of PrP^C^ through activation of ADAM10 could decrease AβO cytotoxicity, we monitored downstream activation of Fyn kinase and ROS production in cells. Fyn kinase is a member of the Src family kinases (SFK), and AβOs activate Fyn kinase by phosphorylation at Tyr-416 (10, 11). Treatment of cells with AβO increased pSFK416 levels 1.3-fold (Fig. 5, *I* and *J*). Pretreatment with acitretin abolished the AβO-mediated increase in pSFK (Fig. 5, *I* and *J*). To monitor the effect of AβO on ROS, cells were primed with menadione to reduce mitochondrial production of NAD(P)H and increase ROS (38). The addition of AβO increased ROS by 1.2-fold in cells expressing PrP^C^ (Fig. 5*K*), but pretreatment with acitretin abolished the increase in ROS mediated by AβO (Fig. 5*K*). Together, these data indicate that promoting the ADAM10-mediated shedding of PrP^C^ blocks both the cellular binding and downstream cytotoxicity of AβO.

### Activation of ADAM10 increases the shedding of PrP^C^ and rescues AβO-mediated increase in ROS in human iPSC-derived cortical neurons

**Figure 6.**
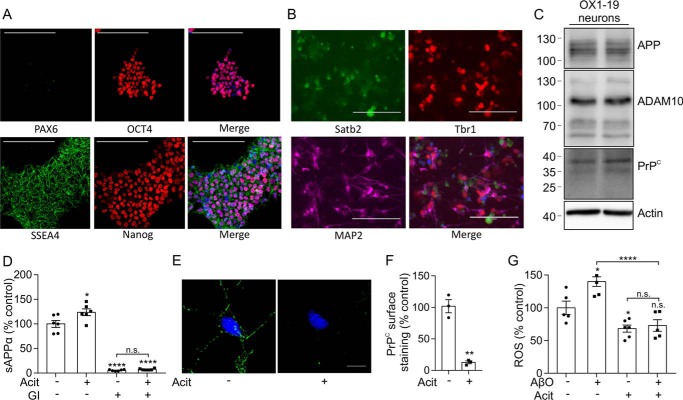
**Activation of ADAM10 by acitretin decreases surface PrP^C^ and decreases AβO toxicity in iPSC-derived neurons.** *A*, immunofluorescence microscopy images showing staining for the pluripotency markers Pax6, Oct4, SSEA-4, and Nanog in permeabilized OX1-19 iPSCs. *Scale bar*, 200 μm. *B*, immunofluorescence microscopy images showing staining for the neuronal markers Satb2, Tbr1, and MAP2 in OX1-19 iPSCs differentiated to cortical neurons at day 60. *Scale bar*, 100 μm. *C*, immunoblots of APP, ADAM10, PrP^C^, and actin in cell lysates from OX1-19 iPSCs differentiated to neurons at day 65. *D*, sAPPα was quantified, using the MSD system, in conditioned media from day 65 OX1-19 cortical neurons incubated with or without GI254023X (*GI*; 10 μm) and with or without acitretin (20 μm) in Opti-MEM for 48 h at 37 °C (*n* = 6). *E*, immunofluorescence microscopy images showing staining for PrP^C^ (*green*) in day 50 OX1-19 cortical neurons incubated with or without acitretin (20 μm) in Opti-MEM for 48 h at 37 °C. *Scale bar*, 5 μm. *F*, quantification of PrP^C^ cell surface staining on nonpermeabilized MAP2-stained neurites, expressed as a percentage of control (*n* = 3). *G*, ROS were measured using the ROS-Glo assay in day 65 OX1-19 cortical neurons with or without acitretin (20 μm) in Opti-MEM for 48 h at 37 °C. Cells were incubated with 1% BSA for 10 min to block the nonspecific binding of AβO and then treated with or without AβO (2.5 μm) for 90 min at 37 °C. Luminescence was measured and is represented as a percentage of control (*n* = 4–7). Statistical analyses were one-way ANOVA with Tukey post hoc correction for multiple-comparison data or independent *t* test with Welch's correction for two-sample comparison. Data are shown as mean ± S.E. (*error bars*). *n.s.*, not significant; *, *p* < 0.05; **, *p* < 0.01; ****, *p* < 0.0001.

To determine whether activation of ADAM10 would lead to enhanced shedding of PrP^C^ and reduce AβO toxicity in human neurons, we used iPSC-derived cortical neurons. The iPSC line OX1-clone 19 (OX1-19) was verified for pluripotency by the presence of Oct4, SSEA-4, and nanog and the absence of PAX6 using immunofluorescence microscopy (Fig. 6*A*). The iPSCs were differentiated into cortical neurons using the method of Shi *et al.* (39). The efficiency of cortical induction was calculated using PAX6 expression (39). This demonstrated that the differentiation efficiency of the OX1-19 and SBAD iPSCs was 92.3 ± 7.6 and 85.9 ± 1.9%, respectively (data described as mean ± S.E. for four cortical inductions of the OX1-19 cell line and for two cortical inductions of the SBAD cell line). The neuronal marker MAP2 was used along with the marker Tbr1 to confirm the presence of secondary progenitor cells and Satb2 to confirm the presence of upper layer neurons (Fig. 6*B*). Immunoblotting of the cortical neurons revealed the presence of APP, ADAM10, and PrP^C^ (Fig. 6*C*).

Acitretin treatment of the iPSC-derived cortical neurons at day 65 increased sAPPα in the media by 1.2-fold (Fig. 6*D*). In the presence of the ADAM10 inhibitor, acitretin failed to induce an increase in the amount of sAPPα in the media (Fig. 6*D*). Treatment with acitretin decreased cell surface PrP^C^ by 87% (Fig. 6, *E* and *F*). AβO treatment increased ROS in the iPSC-derived cortical neurons (Fig. 6*G*), and this increase was blocked by pretreatment of the neurons with acitretin (Fig. 6*G*). These data indicate that acitretin, through increasing ADAM10 activity and the shedding of PrP^C^, blocks the toxicity of AβO in human neurons.

### Activation of ADAM10 increases the neuroprotective sAPPα but does not decrease Aβ production in human neurons

**Figure 7.**
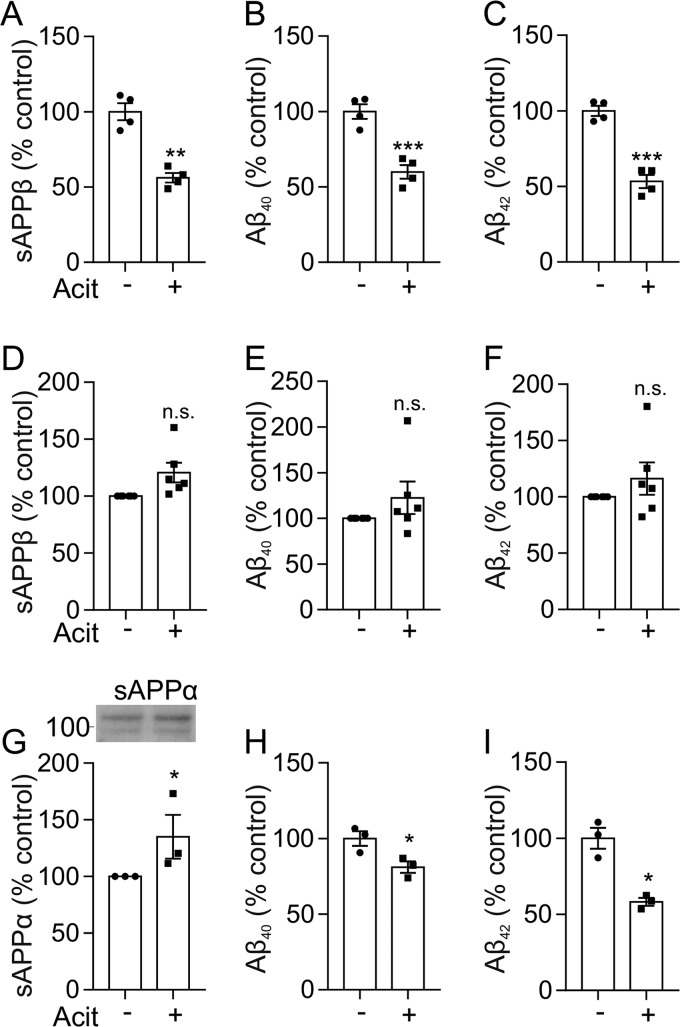
**The effect of acitretin on Aβ production is cell type–dependent.** sAPPβ (*A*), Aβ40 (*B*), and Aβ42 (*C*) were quantified by MSD analysis in conditioned media from SH-SY5Y cells expressing PrP^C^ incubated with or without acitretin (20 μm) in Opti-MEM for 48 h at 37 °C (*n* = 3). sAPPβ (*D*), Aβ40 (*E*), and Aβ42 (*F*) were quantified by MSD analysis in conditioned media from day 65 cortical neurons incubated with or without acitretin (20 μm) in Opti-MEM for 48 h at 37 °C (*n* = 6 differentiations of two iPSC lines, OX1-19 and SBAD). *G–I*, immunoblotting and quantification of sAPPα in concentrated conditioned media (*G*) and quantification of Aβ40 (*H*) and Aβ42 (*I*) by MSD analysis in conditioned media from rat primary hippocampal neurons incubated with or without acitretin (20 μm) in Opti-MEM for 48 h at 37 °C (*n* = 3). Data are expressed as a percentage of control. Statistical analyses were Mann–Whitney *U* test for *n* = 3 and independent *t* test with Welch's correction for *n* > 3. Data are shown as mean ± S.E. (*error bars*). *n.s.*, not significant; *, *p* < 0.05; **, *p* < 0.01; ***, *p* < 0.001.

Activation of ADAM10 has been reported to increase the production of the neuroprotective sAPPα and, reciprocally, decrease Aβ production (30). However, whether this reciprocal relationship occurs in human neurons upon activation of ADAM10 has not been reported. To determine this, we assessed the effect of acitretin on the relative amounts of sAPPα, sAPPβ, and Aβ peptides in both the SH-SY5Y cells and the iPSC-derived neurons. In the SH-SY5Y cells, acitretin caused a significant increase in sAPPα (Fig. 5*C*) and a reciprocal significant decrease in sAPPβ (Fig. 7*A*). The decrease in sAPPβ was paralleled by a decrease in both Aβ40 and Aβ42 following acitretin treatment (Fig. 7, *B* and *C*). However, although acitretin increased sAPPα in the iPSC-derived neurons (Fig. 6*C*), there was no reciprocal decrease in sAPPβ or Aβ (Fig. 7, *D–F*). To ascertain whether this lack of reciprocal effect of acitretin on the nonamyloidogenic and amyloidogenic pathways was a feature of neurons, the effect of acitretin on rat primary hippocampal neurons was investigated. Acitretin treatment of the hippocampal neurons increased the nonamyloidogenic cleavage of APP (Fig. 7*G*) and caused a reciprocal decrease in both Aβ40 and Aβ42 (Fig. 7, *H* and *I*). Thus, in both the SH-SY5Y cells and the rat hippocampal neurons, activation of the nonamyloidogenic processing of APP results in a reciprocal decrease in the amyloidogenic processing pathway, whereas in the human iPSC-derived neurons, no such reciprocal relationship was observed.

## Discussion

Numerous studies have validated PrP^C^ as a key neuronal receptor for AβO and highlighted the intrinsic role it plays in the activation of multiple downstream targets, leading to neuronal impairment in AD (reviewed in Refs. 4 and 5). As cleavage of PrP^C^ by ADAM10 regulates the amount of PrP^C^ at the cell surface (31–33), we hypothesized that modulation of ADAM10 activity, through altering cell surface PrP^C^, would impact AβO binding and toxicity. Here, we show that increasing ADAM10 activity promoted the shedding of cell surface PrP^C^ and, as a result, blocked the binding of AβO to the surface of neurons and decreased their cytotoxicity as measured by activation of Fyn kinase and increase in ROS.

Initially, we used the muscarinic agonist carbachol to promote the shedding of PrP^C^ based on the observation that carbachol promotes the shedding of APP, likely through the activation of ADAM10 (28). Carbachol decreased the amount of PrP^C^ at the cell surface, which resulted in reduced binding of AβO to the cells. Through the use of a selective ADAM10 inhibitor, we show for the first time that carbachol is acting via ADAM10 to promote the shedding of both PrP^C^ and APP. To confirm that ADAM10 was altering AβO binding through modulating cell surface PrP^C^, we used siRNA knockdown to reduce ADAM10, which resulted in increased cell surface presentation of PrP^C^ and a concomitant increase in AβO binding. Although various other proteins have been reported to act as cell surface receptors for AβO (reviewed in Ref. 4), the effect of carbachol activation of ADAM10 on AβO binding was mediated specifically via PrP^C^. This was evidenced by (i) binding being blocked using the 6D11 antibody, whose epitope corresponds to the AβO-binding site on PrP^C^, and (ii) siRNA knockdown of ADAM10 in cells lacking PrP^C^ failing to increase AβO binding. The AβO used here have been well-characterized using biophysical and immunological techniques and correspond to fibrillar oligomers recognized by the conformation-specific OC antibody (11). Such OC-reactive AβO correlated with the onset and severity of AD in human brain (40) and with cognitive decline and tau aggregation and phosphorylation in a transgenic AD mouse model (41). Whether activation of ADAM10 will influence the cellular binding of other oligomeric species of Aβ will depend on whether their receptors are also susceptible to ADAM10-mediated shedding.

In addition to using carbachol to activate ADAM10, we also employed the synthetic retinoid, acitretin, which releases all-*trans*-retinoic acid from cellular retinoic acid–binding proteins. This allows the all-*trans*-retinoic acid to bind to retinoid acid receptor transcription complexes, which in turn bind to retinoid binding sites on the *ADAM10* promoter, leading to increased ADAM10 activity (30). Acitretin has previously been reported to increase the α-secretase cleavage of APP by ADAM10 both in cellular and animal models (30). Similarly, we report that acitretin promoted the shedding of APP and PrP^C^, both in SH-SY5Y cells and iPSC-derived neurons, which was blocked by the ADAM10-specific inhibitor. Thus, using two independently acting activators of ADAM10, we clearly show that promoting the activity of this metalloprotease reduces the cellular binding and cytotoxicity of AβO through modulating cell surface PrP^C^.

ADAM10 promotes the shedding of the ectodomain of PrP^C^ by cleaving the protein after residue 228, close to the site of attachment of the GPI anchor (residue 231) (31). However, it has also been proposed that ADAM10 is responsible for the α-cleavage of PrP^C^ between amino acids 110 and 111 just C-terminal to the AβO-binding domain (residues 95–105) (6, 42), releasing the soluble N1 fragment containing the AβO-binding domain and leaving the cell-associated C1 fragment (35), an action that would also be predicted to reduce AβO binding to the cell surface. The N1 fragment has been shown to bind AβOs and suppress their toxicity in cultured murine hippocampal neurons and in an *in vivo* mouse model of Aβ-induced memory dysfunction, leading to the suggestion that up-regulation of N1 production could act as a cellular mechanism to protect against AβO toxicity (43, 44). Carbachol has previously been reported to increase the α-cleavage of PrP^C^ in HEK293 cells through the action of ADAM17 (45, 46). Here, we show that in the SH-SY5Y cells, which express functional muscarinic receptors (29, 47), the carbachol-stimulated shedding of PrP^C^ and APP is due to activation of ADAM10, not ADAM17. Furthermore, we were unable to detect the soluble N1 fragment in the conditioned medium from the SH-SY5Y cells following carbachol treatment (data not shown). The differences between our work and that previously published (45, 46) may reflect the use of different cell lines in which the relative levels of ADAM10 and ADAM17 differ and/or that in the SH-SY5Y cells, the N1 fragment is rapidly metabolized. However, as activation of ADAM10 in the SH-SY5Y cells did not influence the ratio of full-length PrP^C^ to the C1 fragment, this indicates that the α-cleavage of PrP^C^ is unaltered in our experimental model, ruling out a contribution of α-cleavage of PrP^C^ to the mechanism by which ADAM10 activation blocks AβO binding.

AβOs mediate a range of cellular actions that contribute to their neurotoxicity in AD. Binding of AβO to PrP^C^ leads to activation of Fyn kinase (10, 11), which in turn phosphorylates *N*-methyl-d-aspartate receptors, altering their cell surface distribution (10), and directly phosphorylates tau on Tyr-18 (9). AβOs also induce cytotoxicity through increasing ROS (48), although to date whether this effect is mediated via their binding to PrP^C^ has not been reported. Here, we show that activation of ADAM10 blocked the AβO-dependent activation of Fyn phosphorylation mediated by PrP^C^. Furthermore, and for the first time, we report that in both SH-SY5Y cells and human iPSC-derived neurons, AβO binding to PrP^C^ increases cellular ROS and that this increase in ROS can be blocked by removing cell surface PrP^C^ upon ADAM10 activation. Thus, ADAM10 activation can ameliorate the downstream cytotoxicity induced by AβO binding to PrP^C^ and represents another potential therapeutic approach to disrupt the AβO-PrP^C^ signaling complex in AD.

Genetic analyses of families with late-onset AD revealed two rare mutations (Q170H and R181G) in the pro-domain of ADAM10 that attenuated its α-secretase activity and shifted APP processing toward β-secretase–mediated cleavage with a 2–3-fold increase in Aβ levels, enhanced Aβ plaque load, and reactive gliosis (49, 50). Based on our work with human iPSC-derived neurons, it is likely that these mutations in ADAM10 will also lead to increased cell surface PrP^C^ and enhanced AβO binding and cytotoxicity, which may contribute to the AD phenotype in individuals with such ADAM10 mutations.

The general consensus is that there is competition between the α- and β-secretases for their substrate APP. For example, in murine primary cortical neurons, ADAM10 knockdown increased sAPPβ and Aβ (51), and in human iPSC-derived neurons, inhibition of BACE1 reduced sAPPβ and Aβ and increased sAPPα (25). However, other studies have failed to observe such a reciprocal relationship (52). On the whole, however, activation of ADAM10 has been reported to result in an increase in sAPPα with a reciprocal decrease in sAPPβ and Aβ peptides in various cell and transgenic mouse models (24, 30, 53, 54). For example, in APP_V717I_ transgenic mice, moderate neuronal overexpression of ADAM10 increased the secretion of sAPPα, reduced the formation of Aβ peptides, and prevented their deposition in plaques, whereas expression of mutant catalytically inactive ADAM10, which acts in a dominant negative manner, led to an increase in the number and size of plaques in the double transgenic mice (24). Stimulation of ADAM10 promoter activity with the vitamin A analogue acitretin led to an increase of mature ADAM10 protein that increased the ratio between α- and β-secretase activity in SH-SY5Y cells, whereas intracerebral injection of acitretin in APP/PS-1 transgenic mice led to a reduction of Aβ40 and Aβ42 (30). Thus, we were somewhat surprised that although activating ADAM10 with acitretin led to a reduction in Aβ levels in the SH-SY5Y cells and in the murine hippocampal neurons, no such decrease in Aβ40, Aβ42, or sAPPβ was observed in iPSC-derived neurons from two different individuals, despite there being a similar increase in sAPPα in all three cell models. This result with human neurons is consistent with the result from a pilot clinical trial in which 21 mild to moderate AD patients were treated with acitretin for 4 weeks. In the AD patients, acitretin caused a significant increase in CSF sAPPα when compared with a placebo control group, but there was not a reciprocal significant decrease in sAPPβ, and Aβ42 was unchanged between the two groups (37). Together, these observations suggest that the reciprocal relationship between the nonamyloidogenic and amyloidogenic APP-processing pathways may not hold true in human neurons when α-secretase cleavage is stimulated.

Regardless of whether activation of ADAM10 leads to a decrease in Aβ by shifting the balance of APP processing between the nonamyloidogenic and amyloidogenic pathways, activation of ADAM10 could be beneficial in AD by acting through other mechanisms. Activation of ADAM10 will directly increase sAPPα that has neuroprotective, neurotrophic, and neurogenic properties (55–57), elevates adult neurogenesis in the hippocampus (50), and has been reported to decrease Aβ generation by directly associating with BACE1 and modulating its activity (58). In addition, as we have demonstrated here, activation of ADAM10 will increase the shedding of PrP^C^ to reduce the cellular binding and downstream toxicity of AβO. However, as well as shedding APP and PrP^C^, ADAM10 also proteolytically cleaves more than 90 membrane proteins in the central nervous system, many of which are essential for brain development and normal physiological functions (59, 60). Thus, whether activation of ADAM10 will only have beneficial effects is unclear, a situation that may be exacerbated if activation were to occur over a prolonged period of time as required to treat a chronic condition such as AD.

We have shown that activation of ADAM10 promotes the proteolytic shedding of the AβO receptor PrP^C^ from the surface of human neuroblastoma cells and iPSC-derived neurons. This shedding of PrP^C^ reduces the binding of AβO to cells and blocks their cytotoxicity as monitored by activation of Fyn kinase and increase in ROS. These data provide the first indication that modulating cell surface PrP^C^ may contribute to the therapeutic potential of ADAM10 activation in AD and contribute to the neurodegeneration observed in individuals with mutations in ADAM10. In addition, we report that activation of ADAM10 does not result in a decrease in Aβ levels in the human iPSC-derived neurons, a result consistent with the results from a study following acitretin administration in humans (37) and highlighting that human iPSC-derived neurons are a valuable model system to explore the mechanisms underlying AD.

## Experimental procedures

### Cell culture

SH-SY5Y human neuroblastoma cells were stably transfected with the cDNA encoding murine PrP^C^ containing the 3F4 epitope tag (human M108/M111) as described previously (61). Both untransfected and PrP^C^-expressing SH-SY5Y cells were cultured in Dulbecco's modified Eagle's medium supplemented with 10% (v/v) fetal bovine serum. NB7 human neuroblastoma cells were cultured in RPMI 1640 medium (Sigma) supplemented with 10% (v/v) fetal bovine serum. Cells were maintained in a humidified incubator at 37 °C in a 5% CO_2_, 95% air atmosphere.

### Culture and differentiation of induced pluripotent stem cells

The iPSC lines, OX1-19 (obtained from S. Cowley, University of Oxford) (62) and SBAD03-05 (obtained from StemBANCC) (63) were maintained on Matrigel (BD Biosciences) in mTeSR1 medium (StemCell Technologies) containing 50 units/ml penicillin and 50 μg/ml streptomycin (Life Technologies, Inc.) in a humidified incubator at 37 °C in a 5% CO_2_, 95% air atmosphere. iPSCs were differentiated to cortical neurons as described previously (39), using dual-SMAD inhibition by 1 μm dorsomorphin and 10 μm SB431452 (Tocris). Following successful differentiation, neural progenitor cells were replated on day 35 postinduction at 250,000 cells/well onto polyornithine and laminin-coated (Sigma) 6-well tissue culture plates and cultured until day 65 postinduction with medium changes every 2–3 days. iPSC pluripotency and successful cortical neuron differentiation were confirmed using immunofluorescence microscopy with appropriate markers.

### Preparation of rat primary hippocampal neurons

Primary neurons were prepared from the hippocampi of 1–2-day-old Wistar rats and cultured as described previously (64). Neurons were cultured for 14 days with medium changes every 3–4 days.

### Activation and inhibition of ADAM10

To activate ADAM10, cells were incubated in Opti-MEM containing GlutaMAX (Life Technologies) containing either 20 μm carbachol (Sigma) for 24 h or 20 μm acitretin (Sigma) for 48 h. To inhibit ADAM10, cells were incubated with 10 μm GI254023X (Tocris), a selective ADAM10 inhibitor, for either 24 or 48 h in Opti-MEM. DMSO only–treated cells were used for comparison with treated cells.

### RNAi studies

siRNA specific for human ADAM10 and a nontargeting sequence were obtained as SMARTpools from Dharmacon (Thermo Fisher Scientific). An additional, single siRNA sequence for human ADAM10 was also obtained for verification experiments (Ambion). SH-SY5Y (untransfected or PrP^C^-expressing) cells were seeded into T75 flasks or 24-well plates in routine culture medium and allowed to adhere overnight. The cell monolayers were washed twice with Dulbecco's PBS (DPBS), and a 25 nm (final concentration) SMARTpool siRNA solution was delivered as a complex with DharmaFECT-1 transfection reagent (Dharmacon) in serum-containing DMEM for 48 h. Cells were then washed twice in DPBS prior to incubation in Opti-MEM for 24 h.

### Conditioned medium, cell lysate, and cell membrane preparation

Conditioned medium was harvested, and cell debris was pelleted by centrifugation at 500 × *g* for 5 min. A 1-ml sample of conditioned medium was removed for immunoassay and stored at −20 °C. Remaining conditioned media were concentrated to 100 μl in a Vivapsin 20-ml concentrator (10,000 molecular weight membrane) by centrifugation at 1,900 × *g* for ∼1 h in a bench top centrifuge at 4 °C. Cells were washed in PBS (Lonza), harvested, and pelleted at 1,400 × *g* for 3 min. Cells were lysed on ice for 30 min in radioimmune precipitation assay buffer (50 mm Tris/HCl, 150 mm NaCl, 0.5% (w/v) sodium deoxycholate, 1% (v/v) Nonidet P-40, pH 8.0) containing protease and phosphatase inhibitor mixtures (Roche Diagnostics Ltd.). Cell lysates were clarified by centrifugation at 12,460 × *g* for 10 min at 4 °C, and the clarified lysate was stored at −20 °C before use. For the preparation of membranes, cells were resuspended in 3 ml of 50 mm HEPES, pH 7.5, and sonicated at amplitude 7 μm for 30 s using a Soniprep150 disintegrator. The cell suspension was then centrifuged at 2,500 × *g* for 10 min at 4 °C to pellet cell membranes and nuclei. The supernatant was then centrifuged in a Beckman Coulter Optima at 100,000 × *g* for 1 h at 4 °C. Membranes were resuspended in 50 mm Tris/HCl, pH 7.5, 2 mm EDTA, 150 mm NaCl, and 1% (w/v) CHAPSO. Protein concentration of all samples was determined by a bicinchoninic acid assay.

### Deglycosylation of cell lysate samples

All deglycosylation solutions were purchased from Prozyme. Cell lysates were made up to 100 μg of protein in a 150-μl volume and boiled for 5 min in 20% (v/v) 5× *N*-glycanase reaction buffer and 4% denaturation solution. After cooling, 4% detergent solution was added to each tube. To one of the tubes, 0.5% (v/v) *N*-glycanase (200 milliunits) was added and incubated at 37 °C for 16 h.

### Immunoblotting

Samples were made up in dissociation buffer (1× dissociation buffer (100 mm Tris-HCl, 2% (w/v) SDS, 10% (v/v) glycerol, 100 mm DTT, 0.02% (w/v) bromphenol blue, pH 6.8) and heated at 95 °C for 5 min. Proteins were resolved by SDS-PAGE on 7–17% acrylamide Tris-glycine gels and then transferred to Hybond polyvinylidene difluoride membranes (GE Healthcare). Following electrotransfer, the membranes were blocked for 1 h in PBS with 0.1% Tween 20 (PBST) and 5% (w/v) nonfat milk and then incubated with primary antibody overnight at 4 °C. Antigens were probed using the following primary antibodies: anti-APP (22C11, Millipore), SAF32 (anti-PrP N terminus, Cayman Chemical), 8H4 (anti-PrP residues 175–185), anti-ADAM10 antibody (Abcam), 6E10 (anti-Aβ(1–17), Merck Biosciences), AC15 (anti-β-actin) and synapsin 1 (Sigma), 2B3 (anti-human sAPPα, Immuno-Biological Laboratories), and anti-phospho-Src family kinase (Tyr-416; Cell Signaling Technology). Primary antibodies were detected by incubation with horseradish peroxidase–conjugated secondary antibody, both in PBST containing 2% BSA. Bound horseradish peroxidase conjugates were visualized using the ECL® detection system with a Syngene Gbox XT4 (Syngene). Densitometric analysis was performed using Genetools analysis software (Syngene).

### ImageStream imaging cytometry

SH-SY5Y cells expressing PrP^C^ were incubated in Opti-MEM containing GlutaMAX and either 20 μm carbachol for 24 h or 20 μm acitretin for 48 h. DMSO only–treated cells were used for comparison with treated cells. Cells were collected in PBS without metals and recovered by centrifugation (300 × *g* for 5 min). All subsequent procedures were carried out at 4 °C. Cells were resuspended in blocking buffer (10% donkey serum in PBS containing metals) at 20 × 10^6^ cells/ml and incubated in primary antibody, SAF32, for 1 h before washing twice with PBS and recovering cells by centrifugation. Cells were then resuspended in blocking buffer and incubated with donkey anti-mouse Alexa Fluor® 488 (Invitrogen) for 30 min in the dark. Cells were washed twice in PBS, fixed in 3% (v/v) paraformaldehyde, and then finally resuspended in PBS before analysis using the ImageStreamX MkII imaging cytometer (Amnis). Brightfield, fluorescence, and dark field scatter images were collected at ×40 magnification for 3000 cells over six biological repeats. Cells were identified by the area and aspect ratio parameters. In focus cells were identified as having a gradient root mean square measure of >40. AlexaFluor® 488 emission was generated by a 488-nm laser set to 100 milliwatts in the INSPIRE software (Amnis). Data were analyzed in IDEAS software (Amnis) and exported to Flowjo version 10 (Tree Star, Inc.) to generate overlay histograms.

### Aβ oligomer preparation

Synthetic biotin-Aβ(1–42) containing a 6-carbon linker between the biotin moiety and the N terminus of Aβ was purchased from AnaSpec (San Jose, CA). AβOs were prepared as described previously (11). Briefly, Aβ peptide was dissolved in 1,1,1,3,3,3-hexafluoropropan-2-ol to break down any aggregated material, dried under a stream of N_2_ gas, and stored at −80 °C. Peptide films of biotin-Aβ(1–42) were dissolved in DMSO to 1 mm and then resuspended in Ham's F-12 medium (Lonza) to a final monomer concentration of 100 μm and incubated for 18–24 h at room temperature. The preparation was then centrifuged at 14,000 × *g* for 20 min to pellet out any fibrillar material, and the supernatant was retained as the oligomer preparation.

### Fluorescence microscopy

SH-SY5Y cells were cultured in growth medium to ∼60% confluence on glass coverslips before required incubations (addition of carbachol, acitretin, or GI254023X, as described) or ADAM10 siRNA treatments were carried out. For AβO-binding experiments, cells were incubated with 500 nm AβO (total peptide concentration) diluted in Opti-MEM for 30 min at 37 °C. Where indicated, a 20-min preincubation with or without 10 μg/ml 6D11 antibody (Covance, BioLegend) was carried out before incubation with AβOs. Postincubation, cells were fixed with 4% (v/v) paraformaldehyde and blocked overnight at 4 °C in DPBS with 5% (v/v) fish skin gelatin. Where required, cells were permeabilized in 0.1% Triton X-100 for 10 min at room temperature before blocking. Coverslips were subsequently incubated for 2 h in the same buffer containing primary antibody, washed, and then incubated with the corresponding fluorescently labeled secondary antibody. PrP^C^ was detected using SAF32 antibody followed by donkey anti-mouse AlexaFluor® 488, and MAP2 was detected with anti-MAP2 (Millipore) followed by goat anti-chicken Alexa Fluor 647 Cy5-IgG (Invitrogen) and biotin-Aβ(1–42) (for detection of AβO) using Texas Red–conjugated streptavidin (Invitrogen). Nuclei were counterstained by washing briefly in DAPI stain, and coverslips were mounted onto glass slides using Fluoromount-G (Southern Biotech). Cells were visualized using a DeltaVision optical restoration microscopy system (Applied Precision). Data were collected from 12 0.5-μm-thick optical sections, and three-dimensional data sets were deconvolved using the softWoRx program (Applied Precision). Images analyzed were individual *z*-sections taken from the middle of the data stack, representing a section through the center of the cell. The number of cells analyzed is indicated in individual figure legends. Fluorescence around the cell membrane was quantified using ImageJ software as described previously (65). This was plotted as pixel intensity *versus* distance around the cell using Microsoft Excel, and then the percentage of cell surface with detectable staining was calculated from multiple images.

For the fluorescence microscopy of iPSCs and iPSC-derived cortical neurons, cells were grown on laminin-coated coverslips, fixed with 4% (v/v) paraformaldehyde, and blocked for 4 h at room temperature in DPBS with 10% (v/v) donkey serum. Where required, cells were permeabilized in 0.2% Triton X-100 for 4 min at room temperature before blocking. Coverslips were then incubated with iPSC-specific markers (PAX6, Oct4, SSEA-4, and nanog; Abcam) to check pluripotency and with neuronal markers (Satb2, Tbr1, and MAP2; Abcam) to confirm differentiation. Each primary antibody was detected with the corresponding secondary antibody, Alexa Fluor 488, 594, or 647. DAPI stain was applied, and coverslips were mounted using ProLong Gold containing DAPI (Southern Biotech). Cells were visualized using an EVOS FL cell imaging system (Thermo Fisher Scientific).

### qPCR

SH-SY5Y cells expressing PrP^C^ were incubated in Opti-MEM containing GlutaMAX and 20 μm acitretin for 48 h. DMSO only-treated cells were used for comparison with treated cells. Cells were harvested, and RNA was extracted using the RNeasy plus kit (QIAgen) according to the manufacturer's instructions. cDNA was synthesized using 1 μg of prepared RNA using the iScript cDNA synthesis kit (Bio-Rad) according to the manufacturer's instructions. The mRNA expression level of ADAM10 was analyzed by real-time qPCR using the SYBR Green method (Applied Biosystems) with the sense and antisense primers reported previously (66). Samples were analyzed in triplicate on a Quantstudio 3 (Life Technologies), and relative expression was calculated using ribosomal qPCR as the control.

### Multiplex immunoassay

Aβ40 and Aβ42 were measured using the V-PLEX Aβ peptide panel 1 (6E10) assay (MSD (Meso Scale Discovery), catalog no. K15200E). sAPPα and sAPPβ were measured using the sAPPα/sAPPβ multiplex assay kit (MSD, catalog no. K15120E) according to the manufacturer's instructions. Assay plates were blocked, and conditioned cell medium samples and standards buffered with 500 mm HEPES, pH 7.4, to a final concentration of 50 mm were loaded in duplicate. Following washing and secondary antibody incubation, assays were read using the MESO QUICKPLEX SQ 120 imager and analyzed using MSD Workbench 4.0 software. The protein concentration of the conditioned medium was determined by a bicinchoninic acid assay, and sAPPα, sAPPβ, and Aβ levels were corrected for total protein concentration.

### ROS assay

For the detection of ROS in cells, H_2_O_2_ production was measured with the ROS-Glo^TM^ H_2_O_2_ assay system (Promega). SH-SY5Y cells expressing PrP^C^ were seeded onto black-walled, clear bottom 96-well plates and allowed to adhere overnight. Cells were then incubated with or without 20 μm acitretin diluted in Opti-MEM for 48 h before incubation with or without AβO (500 nm) in the presence of 10 μm menadione (Sigma) and the H_2_O_2_ substrate at 37 °C for 90 min. ROS-Glo detection solution and signal enhancer were then added, and, after a 20-min incubation at room temperature, luminescence was measured with a Synergy HT Bio-Tek fluorimeter using Gen5 software. For experiments with cortical neurons, the above method was used with the following modifications. Cells were plated at day 35 and cultured until day 65. Following acitretin incubation, 1% BSA was added to cell cultures for 10 min to block the nonspecific binding of AβO to the laminin coating on the plates prior to incubation with 2.5 μm AβOs.

### Statistical analysis

Data were analyzed as stated in the figure legends, and *n* numbers are specified. For statistical analysis, data were analyzed using GraphPad Prism version 7.00. A normal distribution was assumed for all cell data as mean values were recorded from a population of cells (based on the assumption that cells from a clonal population will respond in a similar manner), and therefore parametric analyses were performed. For comparison between two data sets, an independent *t* test was applied with Welch's correction (equal S.D. values not assumed). For the rat primary hippocampal neuron data, a Mann–Whitney *U* test was used to compare between groups, as a normal distribution could not be assumed or determined from the sample size. For multiple comparisons, a one-way ANOVA with Tukey's post hoc correction for pairwise comparisons was used. Data are shown as mean ± S.E., and *p* < 0.05 was considered significant. Levels of significance are defined in the figure legends.

## Author contributions

H. H. J.-G., K. A. B. K., and N. M. H. designed the study. H. H. J.-G., N. J. C., H. A. R., K. F., A. C. J., G. J. H., J. B., and K. A. B. K. performed the research. S. A. C., S. C., and M. Z. C. provided reagents. H. H. J. G., N. J. C., K. F., G. J. H., K. A. B. K., and N. M. H. analyzed the data. H. H. J.-G., K. A. B. K., and N. M. H. wrote the paper. All authors read and approved the final manuscript.

## Footnotes

3 The abbreviations used are:

AD: Alzheimer's disease
Aβ: amyloid-β
AβO: amyloid-β oligomer
APP: amyloid precursor protein
ADAM10: a disintegrin and metalloprotease 10
BACE1: β-site APP cleaving enzyme 1
iPSC: induced pluripotent stem cell
PrP^C^: cellular prion protein
ROS: reactive oxygen species
sAPPα: soluble APP fragment produced by α-secretase cleavage
sAPPβ: soluble sAPPβ produced by β-secretase cleavage
GPI: glycosylphosphatidylinositol
SFK: Src family kinase(s)
DPBS: Dulbecco's PBS
DAPI: 4′,6-diamidino-2-phenylindole
qPCR: quantitative PCR
ANOVA: analysis of variance
CHAPSO: 3-[(3-cholamidopropyl)dimethylammonio]-2-hydroxy-1-propanesulfonic acid.
